# Position of Hungarian Merino among other Merinos, within-breed genetic similarity network and markers associated with daily weight gain

**DOI:** 10.5713/ab.21.0459

**Published:** 2022-06-24

**Authors:** Attila Zsolnai, István Egerszegi, László Rózsa, Dávid Mezőszentgyörgyi, István Anton

**Affiliations:** 1 Department of Animal Breeding, Institute of Animal Science, Hungarian University of Agriculture and Life Sciences, Kaposvár Campus, Herceghalom, 2053, Hungary; 2 National Centre for Biodiversity and Gene Conservation, Gödöllő, 2100, Hungary; 3 Department of Animal Husbandry Technology and Animal Welfare, Institute of Animal Science, Hungarian University of Agriculture and Life Sciences, Kaposvár Campus, Gödöllő, 2100, Hungary; 4 Hungarian University of Agriculture and Life Sciences, Georgikon Campus, Keszthely, 8360, Hungary

**Keywords:** Genome-wide Association (GWA), Network, Sheep, Single Nucleotide Polymorphism (SNP), Position, Weight Gain

## Abstract

**Objective:**

In this study, we aimed to position the Hungarian Merino among other Merino-derived sheep breeds, explore the characteristics of our sampled animals' genetic similarity network within the breed, and highlight single nucleotide polymorphisms (SNPs) associated with daily weight-gain.

**Methods:**

Hungarian Merino (n = 138) was genotyped on Ovine SNP50 Bead Chip (Illumina, San Diego, CA, USA) and positioned among 30 Merino and Merino-derived breeds (n = 555). Population characteristics were obtained via PLINK, SVS, Admixture, and Treemix software, within-breed network was analysed with python networkx 2.3 library. Daily weight gain of Hungarian Merino was standardised to 60 days and was collected from the database of the Association of Hungarian Sheep and Goat Breeders. For the identification of loci associated with daily weight gain, a multi-locus mixed-model was used.

**Results:**

Supporting the breed's written history, the closest breeds to Hungarian Merino were Estremadura and Rambouillet (pairwise F_ST_ values are 0.035 and 0.036, respectively). Among Hungarian Merino, a highly centralised connectedness has been revealed by network analysis of pairwise values of identity-by-state, where the animal in the central node had a betweenness centrality value equal to 0.936. Probing of daily weight gain against the SNP data of Hungarian Merinos revealed five associated loci. Two of them, OAR8_17854216.1 and s42441.1 on chromosome 8 and 9 (−log_10_P>22, false discovery rate<5.5e–20) and one locus on chromosome 20, s28948.1 (−log_10_P = 13.46, false discovery rate = 4.1e–11), were close to the markers reported in other breeds concerning daily weight gain, six-month weight, and post-weaning gain.

**Conclusion:**

The position of Hungarian Merino among other Merino breeds has been determined. We have described the similarity network of the individuals to be applied in breeding practices and highlighted several markers useful for elevating the daily weight gain of Hungarian Merino.

## INTRODUCTION

Merino is a globally distributed fine-wool sheep breed representing about a quarter of the global sheep population [1]. The appearance of the first European fine-wool sheep in the southern region of Spain and Italy dates back to the 1st century BCE. These animals were cross-bred with local and imported Arabian sheep [2], likely resulting in a breed that can be considered an early ancestor of the Merino. Ciani et al [3] lean towards the opinion that the Merino breed has an Iberian origin with other stock contributions from the Mediterranean region. Until 1760, the export sale of Merinos from Spain was prohibited. Thenceforward, small flocks were exported to several European countries [4]. The first flock arrived in Hungary in 1765 due to a decree emitted by Empress Maria Theresa of Austria [5]. This importation was followed by two others in 1773 and 1775 [6]. The last Merino export from Spain to Hungary was recorded in 1802.

The Industrial Revolution in Britain led to considerable improvements in weaving efficiency. The development of the wool textile industry resulted in an increasing global demand for wool. The selection process for wool quantity and quality resulted in many types of Merino sheep worldwide [7]. The Hungarian Combed Merino breed was developed from imported Merinos, the local Racka breed, and German domestic sheep [8]. Importation of Rambouillet sheep from France (1816) and North-German Combed sheep has also contributed to the development of the Hungarian breed [6]. The first Hungarian herd book was introduced in 1859, in this period, Merino and Merino-derived sheep reached 31.5% of the population among Hungarian sheep [4].

At the end of the 19th century, in spite of the Western European meat-breeding trend, wool was still the main production objective in Hungary [9]. After World Wars I and II, crossbreeding resulted in a fine and short-wool population, which became more homogeneous by breeding Précoce and Rambouillet rams [6,9]. In the 1950s, the breed was reshaped by fine-wool variants from the former Soviet Union (Askanian, Caucasian, Stavropol, and Grozny Merinos). This decision led to considerable increases in the body mass and the wool production capabilities of the breed [10]. In the 1960s, French Précoce and German Mutton Merinos were imported to Hungary to improve the breed's meat-production potential. In the 1970s through 1980s, several attempts were made to improve the production parameters and prolificacy of Merino flocks. For that very reason, Kent, Corriedale, and Australian Booroola Merinos were introduced into Hungary [11]. These crosses also breed better muscularity and wool quality [1,10].

In the 1980s, wool production was pushed into the background, and Hungarian Merino was considered a dual-purpose (meat and milk producing) breed [12]. A drastic change in agricultural subsidies followed the democratic transformation of the former socialist regime in 1989. During this period, exported lamb became the main product of sheep husbandry, and the average daily weight gain of lambs was about 250 g/d [13].

In the 2010s, due to considerable declines in breeding activity, Hungarian Merinos became classified as endangered. Subsequently, the Hungarian government began to provide subsidies for the conservation of genetic resources as part of the coordinated breeding program. In 2020, the Hungarian Merino population amounted to 8,167 ewes. At present, only 11 Hungarian genealogical lines are noted [1] out of the 39 recorded in 2004.

We had three aims in this study: i) Since the genetic position of the Hungarian Merino is not known based on single nucleotide polymorphism (SNP) markers, our samples were compared to the database of Ciani et al [3] to determine the genetic position of Hungarian Merinos among other Merino-derived sheep breeds. ii) However, there are publications considering the gene network [14] or genetic connections among the breeds [15], we wanted to explore the characteristics of our sampled animals' genetic similarity network within the breed to increase the ability of sheep breeders (including those keeping other sheep or non-sheep populations in the world) to easily adopt decisions on an individual level. iii) Since nowadays the most important parameter of the Hungarian sheep-breeding industry is the weight-gain of lambs, we decided to deliver markers suitable to improving the daily weight-gain of Hungarian Merino lambs. Our investigation overlaps other activities like investigating growth and meat production traits in several other sheep breeds [16–19] and highlights regions in the sheep genome that are usable not only for Hungarian Merino but for other breeds as well.

## MATERIALS AND METHODS

Approval from the animal research ethics committee was not necessary as the ear tag application is part of the routine breeding procedure.

### Animals, genotyping, and quality control

The sampling locations covered Hungary, which has a quite uniform, continental climate. One hundred thirty-eight Hungarian Merino ewes were included in this study. They were born in different years spanning 10 years. Their daily weight gain was standardised to 60 days as follows: 60 BM_30–80_/d, where BM_30–80_ is the body mass of the lamb measured between 30 and 80 days of age, d is the actual number of days after birth on the day of the measurement.

Ear cartilage samples were collected by the TypiFix (Agrobiogen GmBH, Hilgertshausen, Germany) tissue sampling system [20] during routine ear tag application. For comparative studies, we used the database provided by Ciani et al [3]. SNP typing of Hungarian Merinos was performed on Ovine SNP50 Bead Chip (Illumina, San Diego, USA) by Neogen Corporation (Ayr, UK).

Quality control of data included filtering for mapped and polymorphic markers (minor allele frequency>0.05) with a call rate higher than 0.95. Duplicated samples were not detected (identical by state value, identical by state (IBS)>0.95). Call rates of the genotypes were over 0.95 for all Hungarian Merino samples. The matched database, which included both the Sheep Consortium [3] and Hungarian data, contained a total of 31 breeds, 693 animals (Table 1), and 22,265 SNPs.

In case of within-breed network and association analyses 46,906 SNPs were kept after data filtering.

### Population genetics analyses

Determination of observed and expected heterozygosity (Ho, He), subsequent calculation of inbreeding coefficient (F_IS_), genetic distance (F_ST_), and principal component analysis (PCA) were performed by SNP & Variation Suite 8.1.1 program (GoldenHelix, Bozeman, MT, USA). The inbreeding coefficient is the (observed - expected number of homozygous markers)/(number of genotyped markers - expected number of homozygous markers). Expected number of homozygous markers $=\Sigma_{j=1}^{L}(1-2p_{j}q_{j}\frac{T_{j}}{\left(T_{j}-1\right)})$, where L is the number of genotyped markers, p and q are the frequency of the alleles, and T is twice the number of non-missing genotypes for marker j. The algorithm for estimation of F_ST_ is explained in Weir and Cockerham [21]. In PCA, the top components are determined from the standardised relationship matrix as described by Patterson et al [22].

Admixture software v.1.3 has been used with --cv option for determining the most probable cluster number (K) from the value of cross-validation error in each Ks [23]. Before analysis, genotypes were transformed by PLINK using --recode12 switch.

Treemix 1.13 [24] software has been applied to calculate a dendrogram; block size was 500, the number of repetitions was 1,000. Bootstrap values were determined by the Phylip 3.697 program package [25].

Network analysis concerning the relation of the Hungarian Merinos was based on the identity-by-state value of pairs calculated by SVS program (GoldenHelix, USA).

Identity-by-state pairwise distance between any two individuals is ([number of markers sharing two alleles+0.5× number of markers sharing one allele]/number of markers).

### Within-breed network and association analyses

Based on IBS values, the net properties of the Hungarian Merino samples, such as diameter and betweenness centrality, were calculated and visualised by Python 3.6 software using the libraries networkx 2.3 and matplotlib 3.1.1. Diameter is the largest distance observed between the pairs of nodes or the distance between the two furthest nodes [26]. Betweenness centrality of a given animal/node $=\Sigma_{s\neq v\neq t}^{v}\frac{\sigma_{st|\text{v}}}{\sigma_{st}}$, where v is the number of nodes, σ_st_ is the total number of shortest paths from node s to node t and σ_st_ is the number of those paths that pass through the node v. The strength of the connections (or weight of the edges) is defined by the IBS value of the pairs. IBS values above the 85 percentile were visualised by thick black lines.

For the identification of loci associated with daily weight gain, a multi-locus mixed-model was used [27]. The model was y = Xβ+Zu+e, where y is a continuous variable, the daily weight gain, X is the matrix of fixed effects composed of 46,906 SNPs and covariates (date and place of birth), Z is the incidence matrix relating random effects to each individual, which becomes an identity matrix, because one phenotypic measurement is performed per individual [28]. e stands for the residual effects, and β and u are vectors representing coefficients of fixed and random effects. For further details on mixed linear models, see the link [29].

## RESULTS

### Diversity and structure of populations

Observed heterozygosity ranged from 0.320 to 0.392, slightly below the expected value of 0.397. Based on the observed and expected heterozygosity values, the inbreeding coefficient of breeds ranged from 0.013 to 0.193 (Table 1). Cordoba Spanish Merino was an outlier with its highest F_IS_ value (0.341), whereas, in Hungarian Merino, this value was 0.054. Pairwise values of F_ST_ calculation fell in the range of 0.004 to 0.313 (Supplementary Table S1). F_ST_ values of Hungarian Merino relative to the other breeds were between 0.035 and 0.184 (Table 2).

**Figure 1:**
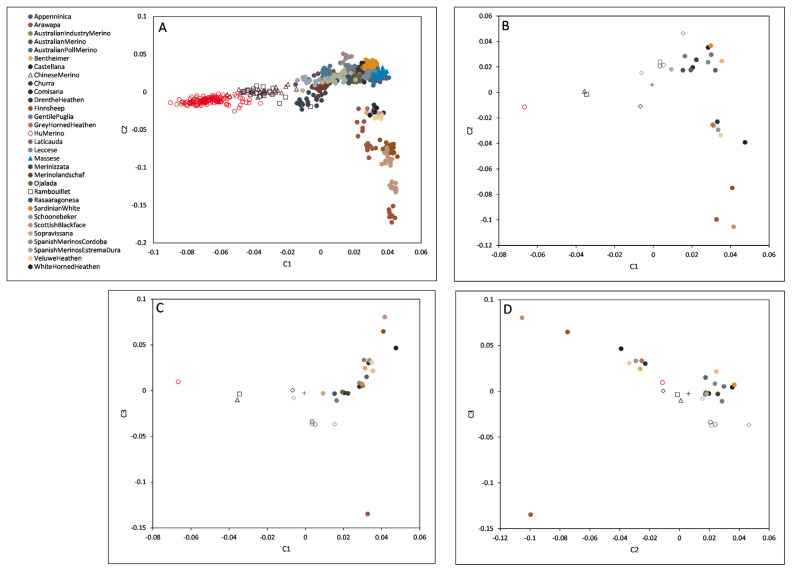
(A) Principal component analysis displaying the relationship between animals coloured by their corresponding breed, see colour codes beside this plot. (B)–(D) Means of the animals on part A belonging to different breeds. C1 = 1st component (eigenvalue = 6.969), C2 = 2nd component (eigenvalue = 3.432), C3 = 3rd component (eigenvalue = 3.304).

Principal component analysis based on pairwise identity-by-state of the animals (Figure 1A–D) placed the Hungarian Merino population on the outer rim of the investigated populations, close to Rambouillet and Chinese Merino.

**Figure 2:**
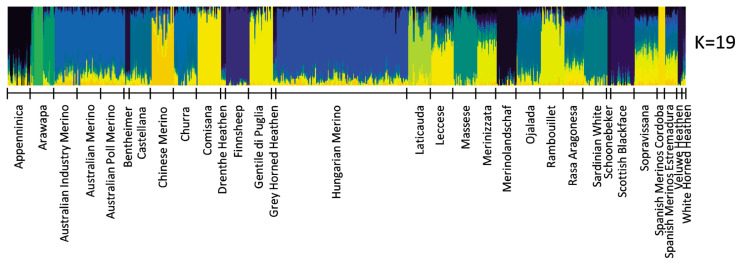
Admixture results concerning the most probably K value.

**Figure 3:**
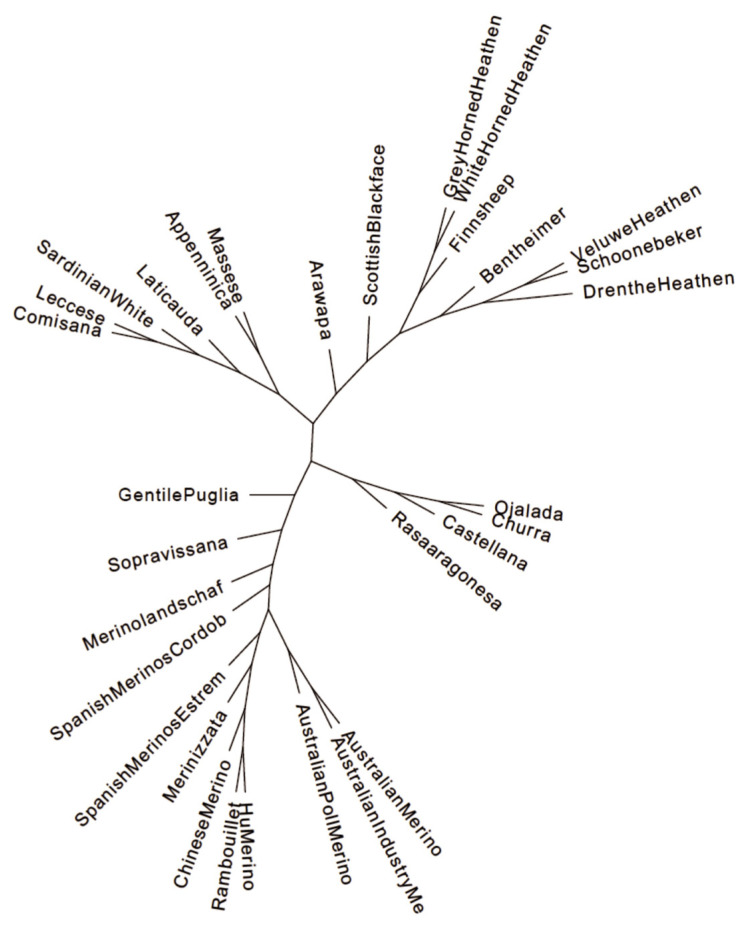
Phylogenetic tree of Merino and Merino-derived breeds constructed with the TreeMix and Phylip programs.

The most probable cluster number of the 31 breeds produced by the admixture analysis was nineteen (K = 19, Figure 2). When K was equal to the number of analysed breeds, most of the Hungarian Merinos displayed admixture with Rambouillet (Supplementary Figure S1). On the dendrogram of the breeds, Hungarian Merino was placed on a common branch with Rambouillet, Chinese Merino, Merinizzata, and Estremadura Spanish Merino (Figure 3; Supplementary Figure S2).

### Identity-by-state network and markers associated with daily weight gain

**Figure 4:**
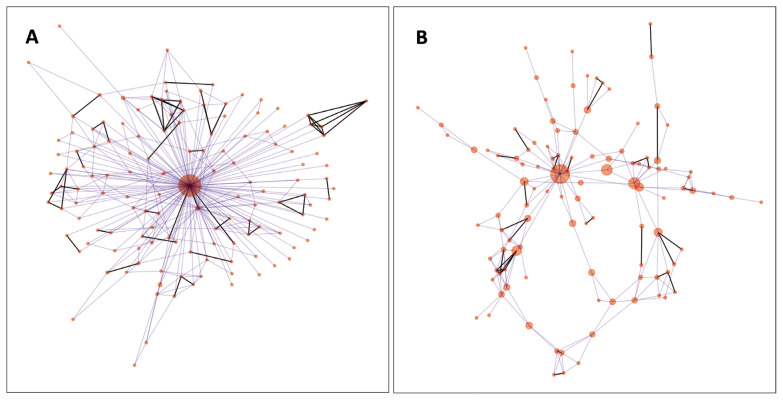
(A–B) Network representation of the Hungarian Merinos based on their pairwise identity-by-state values. The animals with the highest betweenness centrality values, depicted by larger red dots, carry sets of genetic material whose different subsets can be found in other individuals connected by lines between the nodes/animals. (B) Network of individuals after removing the central animal seen on plot A. The highest betweenness centrality values are 0.936 and 0.744 on plot A and B., respectively.

For visualisation of the genetic net of the individuals, IBS values (edges) above 0.73 have been maintained. In the analysed population, this value provided an interconnected network of all nodes (individuals). The number of edges was 341, the average degree was 5.014, net diameter was 4 (Figure 4A). The five highest betweenness centrality values were 0.936, 0.035, 0.034, 0.029, and 0.023. When the animal having the highest betweenness centrality (0.936) was removed, the interconnected network contained 108 animals and 199 edges (Figure 4B). In this case, average degree became 3.682, and the diameter of the net expanded to 10. The highest five betweenness centrality values were 0.744, 0.261, 0.235, 0.185, and 0.141.

The values of daily weight gain ranged from 261.6 to 494.7 grams. In multi-locus mixed-model analysis, several loci were identified to be associated with the daily weight gain (Table 3). The top hits were five in number (−log_10_P>10) on chromosome 2, 3, 8, 9, and 20 with false discovery rates lower than 4.1e–11.

## DISCUSSION

F_IS_ value calculated from observed and expected heterozygosity of Hungarian Merinos is below 0.055. Ten out of the thirty-one studied breeds have small F_IS_ values, indicating that there are not considerably more inbred animals than expected.

We have found that the calculated genetic distances and the position of Hungarian Merinos in PCA and on the phylogenetic tree support the contribution of Rambouillet imported twice [6], to our breed. Similar positions of the Chinese Merino and Rambouillet on the PCA plot and the dendrogram indicate a close relationship between the two breeds. As for the Spanish breeds presented in the database, Estremadura Merino is the closest to Hungarian Merino, presenting its strongest influence on the breed (Table 2). Pairwise F_ST_ value of the Spanish Cordoba and Hungarian Merino breed was the highest (F_ST_ = 0.184), ruling out the Cordoba breed as an origin breed of Hungarian Merino. The phylogenetic study placed Estremadura Merino the fourth closest to Hungarian Merino, while the Rambouillet breed remained the closest (Supplementary Figure S2).

In Admixture analysis, the most probable cluster number (K) was 19 (Figure 2; Supplementary Figure S1); however, the Hungarian Merino’s admixture with Rambouillet was seen at K = 29 and 31 (Supplementary Figure S1).

Network analysis of the individuals revealed (Figure 4A) a spoked wheel structure [26], which is probably induced by the extensive usage of preferable individuals. When the animal with the highest betweenness centrality in the genetic similarity net was removed, the wheel structure disappeared, and more details were revealed. In Figure 4B, we can identify other animals which have strong influences on the breed composition relative to the others. Based on network data, breeders can select suitable animals for optimal mating schemes. Visualisation of edge strengths (a.k.a. pairwise IBS values between the animals) promotes easy recognition of closely related animals (Figure 4). In this way, deterioration of heterozygosity parameters, inbreeding depression, and declination of economic performance can be avoided. Improvement of the visualisation of genetic similarities can further enhance the applicability of this analysis. Improvements could be achieved by pop-up windows carrying cross-references to the breeders, farms, age, ancestors, or by time-lapse interpretation of the connections as members of the populations are changing via newborn and culled animals.

Concerning daily weight gain, we have found five highly correlated loci (Table 3) using 138 animals. However, the sample size is lower than that used by Zhang et al [16] or Lu et al [18], comparing our highly correlated loci with markers revealed by other teams on similar data hints that the main players in a multigenic trait could be exposed with 100+ animals as well. Two of our five markers (OAR8_17854216.1 on chromosome 8 and s42441.1 on chromosome 9) were in close vicinity, −1.3 and +4.8 million bases, respectively, to the top findings of Zhang et al [16]. The reported markers were associated with daily weight gain, six-month weight, and post-weaning weight. A third marker (s28948.1) we highlighted was 11.7, and 1.5 million bases further away from a post-weaning gain [16] and a weaning weight [18] associated marker, respectively. The close vicinity of the above mentioned three markers to those identified by other teams [16,18] might be explained by the high similarity of Hungarian and Chinese Merino revealed by principal components (Figure 1), pairwise genetic distance (Table 2; Supplementary Table S1), and dendrogram (Figure 3; Supplementary Figure S2). Our markers on chromosome 2 and 3, OAR2_137539806.1 and OAR3_188812728.1, or their neighbouring markers closer than twelve million base pairs, were not reported elsewhere related to weight-gain properties.

This study gives insight into the overall genetic state of the Hungarian Merino in contrast to other breeds. The results might be useful for other research conducted on a wider set of Merino breeds.

The within-breed network analysis of the animals based on their genetic similarity and its visualisation could provide an intuitive tool to the breeders of any species to easily have a glimpse of the genetic structure of the population and the most important animals. Later on, when more within-breed structures could be analysed and compared, we could dissect different breeding practices among different breeders or countries, giving further insight into the influence of the breeding policy on the structure of a given breed.

Some of the loci associated with a standardized daily weight gain were very close to the markers presented in other studies on 2.4 and 3.4 higher number of animals (Table 3). Application of these markers could improve the daily weight-gain of lambs, which is important as lambs production provides a considerable income for the breeders.

## Figures and Tables

**Table 1:** Observed, expected heterozygosities (Ho, He) and inbreeding coefficients (F_IS_) calculated on the 31 sheep populations

Breed	Number of samples	Ho	He	F_IS_
Rasa Aragonesa	20	0.392	0.397	0.013
Castellana	22	0.386	0.397	0.028
Ojalada	24	0.383	0.397	0.035
Spanish Merinos Estremadura	13	0.381	0.397	0.041
Comisana	24	0.379	0.397	0.046
Australian Poll Merino	24	0.377	0.397	0.051
Australian Industry Merino	24	0.377	0.397	0.051
Appenninica	24	0.376	0.397	0.053
Merinizzata	20	0.376	0.397	0.054
Hungarian Merino	138	0.376	0.397	0.054
Chinese Merino	23	0.373	0.397	0.061
Sopravissana	24	0.373	0.397	0.062
Churra	24	0.371	0.397	0.065
Laticauda	24	0.371	0.397	0.067
Scottish Blackface	24	0.370	0.397	0.069
Merinolandschaf	21	0.369	0.397	0.070
Gentile di Puglia	24	0.369	0.397	0.071
Australian Merino	24	0.365	0.397	0.080
Sardinian White	24	0.364	0.397	0.083
Massese	24	0.361	0.397	0.090
Leccese	23	0.356	0.397	0.103
Rambouillet	24	0.356	0.397	0.105
Veluwe Heathen	5	0.355	0.397	0.107
Drenthe Heathen	5	0.350	0.397	0.118
Finnsheep	24	0.348	0.397	0.123
Bentheimer	5	0.341	0.397	0.141
Schoonebeker	4	0.324	0.397	0.183
White Horned Heathen	3	0.324	0.397	0.184
Arawapa	24	0.323	0.397	0.186
Grey Horned Heathen	4	0.320	0.397	0.193
Spanish Merinos Cordoba	7	0.262	0.397	0.341

**Table 2:** Pairwise coefficients of genetic differentiation (F_ST_) values between Hungarian Merino and the investigated Merino breeds

Breeds	Hungarian Merino
Hungarian Merino	0.000
Spanish Merinos Estremadura	0.035
Rambouillet	0.036
Rasa Aragonesa	0.038
Chinese Merino	0.040
Sopravissana	0.042
Merinizzata	0.043
Australian Merino	0.045
Australian Industry Merino	0.047
Australian Poll Merino	0.050
Ojalada	0.050
Castellana	0.053
Merinolandschaf	0.055
Leccese	0.058
Churra	0.063
Laticauda	0.064
Comisana	0.065
Gentile di Puglia	0.068
Appenninica	0.071
Sardinian White	0.076
Massese	0.080
White Horned Heathen	0.087
Finnsheep	0.094
Arawapa	0.098
Drenthe Heathen	0.098
Scottish Blackface	0.099
Bentheimer	0.100
Veluwe Heathen	0.103
Schoonebeker	0.117
Grey Horned Heathen	0.124
Spanish Merinos Cordoba	0.184

For values and heat-map of all pairwise F_ST_ values, see Supplementary Table S1.

**Table 3:** List of loci associated with daily weight-gain of Hungarian Merinos

Marker name	Chr:position	−log_10_P^1)^	FDR	ASE	Marker name or Chr:position (investigated trait); publication n^2)^	Delta bp^3)^
OAR2_137539806.1	2:129172257	19.87	1.6e–17	0.506±0.021	2:206466290 (adult weight); Lu et al [18]; 460	77,294,034^*^
OAR3_188812728.1	3:175738965	17.41	4.6e–15	0.506±0.026	3:118519704 (weaning weight); Lu et al [18]; 460	−57,219,261^*^
OAR8_17854216.1	8:15960500	22.70	2.4e–20	0.527±0.017	OAR8_16297646.1 (daily weight gain, 6-month weight); Zhang et al [16]; 329	−1,302,115
s42441.1	9:81626179	23.36	5.5e–21	0.505±0.016	OAR9_91721507.1 (post-weaning gain); Zhang et al [16]; 329	4,779,602
s28948.1	20:27936473	13.46	4.1e–11	−0.333±0.024	s72649.1 (post-weaning gain); Zhang et al [16]; 329 20:26410829 (weaning weight); Lu et al [18]; 460	−11,699,128 −1,525,644^*^

Chr, chromosome number; FDR, false discovery rate; ASE, allele substitution effect and its standard error.

1) −log_10_P = −log_10_ transformed p values obtained by multi-locus mixed-model algorithm.

2) n = number of animals in the referred literature.

3) delta bp = distance of the markers found by Zhang et al [16] or Lu et al [18] relative to the markers found in this study.

* positions of OAR2_137539806.1, OAR3_188812728.1 and s28948.1 were adjusted to Oar4.0 coordinates to make it comparable to the work Lu et al [18].

## SUPPLEMENTARY MATERIAL

**Supplementary Table S1**. Pairwise values of F_ST_ and their heat map

**Supplementary Figure S1**. Admixture analysis of 31 Merino-derived breeds and for a range of K-values (K = 2 to 31) and cross-validation error at different K values. Each individual is represented by a single column divided into K coloured segments, where K is the number of assumed clusters.

**Supplementary Figure S2**. UPGMA tree of Nei’s genetic distance sheep breeds. Bootstrap values are indicated on the nodes.
