# Supplementary material for: Position of Hungarian Merino among other Merinos, within-breed genetic similarity network and markers associated with daily weight gain
Source: Anim Biosci. 2022 Jun 24;36(1):10–8. doi: 10.5713/ab.21.0459 (PMC9834658; doi:10.5713/ab.21.0459)
Supplement: Supplementary file 1 [file ab-21-0459-suppl1.pdf]

SUPPLEMENTARY MATERIAL

Position of Hungarian Merino among other Merinos, within-breed genetic similarity network and markers associated with daily weight gain.

A. Zsolnai, I. Egerszegi, L. Rózsa, D. Mezőszentgyörgyi, I. Anton

Animal Bioscience

SupplementaryTable S1 Pairwise values of F_{ST} and their heat map

Breeds	Appenninica	Arawapa	AustralianIndustryMerino	AustralianMerino	AustralianPollMerino	Bentheimer	Castellana	ChineseMerino	Churra	Comisana	DrentheHeathen	Finnsheep	GentilePuglia	GreyHornedHeathen	HungarianMerino	Laticauda	Leccese	Massese	Merinizzata	Merinolandschaf	Ojalada	Rambouillet	Rasaaragonesa	SardinianWhite	Schoonebeker	ScottishBlackface	Sopravissana	SpanishMerinosCordoba	SpanishMerinosEstremaDura	VeluweHeathen	WhiteHornedHeathen
Appenninica	0	0.099	0.057	0.055	0.060	0.095	0.051	0.078	0.062	0.058	0.093	0.086	0.070	0.122	0.071	0.052	0.045	0.067	0.057	0.067	0.048	0.073	0.038	0.070	0.114	0.095	0.044	0.202	0.052	0.097	0.081
Arawapa	0.099	0	0.081	0.080	0.086	0.127	0.085	0.104	0.096	0.097	0.128	0.117	0.104	0.156	0.098	0.093	0.088	0.111	0.086	0.099	0.082	0.102	0.072	0.109	0.147	0.121	0.081	0.229	0.084	0.128	0.115
AustralianIndustryMerino	0.057	0.081	0	0.004	0.012	0.087	0.038	0.049	0.050	0.052	0.085	0.082	0.057	0.112	0.047	0.049	0.044	0.067	0.045	0.052	0.036	0.049	0.024	0.065	0.103	0.091	0.033	0.183	0.031	0.089	0.072
AustralianMerino	0.055	0.080	0.004	0	0.014	0.084	0.037	0.046	0.048	0.050	0.081	0.080	0.055	0.109	0.045	0.047	0.042	0.066	0.043	0.049	0.035	0.045	0.022	0.062	0.100	0.088	0.030	0.180	0.028	0.086	0.067
AustralianPollMerino	0.060	0.086	0.012	0.014	0	0.091	0.042	0.052	0.054	0.055	0.089	0.086	0.060	0.116	0.050	0.052	0.048	0.071	0.048	0.054	0.040	0.051	0.028	0.068	0.107	0.095	0.036	0.187	0.034	0.094	0.075
Bentheimer	0.095	0.127	0.087	0.084	0.091	0	0.085	0.112	0.094	0.094	0.104	0.106	0.109	0.162	0.100	0.091	0.081	0.110	0.088	0.099	0.081	0.103	0.069	0.109	0.103	0.119	0.079	0.276	0.085	0.099	0.107
Castellana	0.051	0.085	0.038	0.037	0.042	0.085	0	0.059	0.040	0.045	0.081	0.078	0.056	0.109	0.053	0.042	0.038	0.062	0.046	0.051	0.028	0.055	0.018	0.059	0.100	0.084	0.031	0.183	0.032	0.086	0.067
ChineseMerino	0.078	0.104	0.049	0.046	0.052	0.112	0.059	0	0.069	0.071	0.109	0.100	0.075	0.138	0.040	0.069	0.064	0.089	0.050	0.063	0.056	0.044	0.043	0.085	0.128	0.108	0.047	0.205	0.043	0.113	0.097
Churra	0.062	0.096	0.050	0.048	0.054	0.094	0.040	0.069	0	0.055	0.091	0.089	0.067	0.122	0.063	0.053	0.049	0.072	0.057	0.062	0.037	0.065	0.029	0.067	0.112	0.095	0.042	0.192	0.044	0.097	0.078
Comisana	0.058	0.097	0.052	0.050	0.055	0.094	0.045	0.071	0.055	0	0.091	0.088	0.067	0.120	0.065	0.044	0.037	0.068	0.057	0.065	0.043	0.069	0.032	0.061	0.111	0.097	0.042	0.194	0.046	0.097	0.078
DrentheHeathen	0.093	0.128	0.085	0.081	0.089	0.104	0.081	0.109	0.091	0.091	0	0.103	0.104	0.155	0.098	0.088	0.079	0.105	0.088	0.098	0.077	0.103	0.066	0.106	0.106	0.115	0.076	0.272	0.083	0.093	0.102
Finnsheep	0.086	0.117	0.082	0.080	0.086	0.106	0.078	0.100	0.089	0.088	0.103	0	0.099	0.125	0.094	0.083	0.078	0.098	0.082	0.093	0.076	0.097	0.066	0.101	0.122	0.100	0.075	0.226	0.080	0.104	0.082
GentilePuglia	0.070	0.104	0.057	0.055	0.060	0.109	0.056	0.075	0.067	0.067	0.104	0.099	0	0.135	0.068	0.062	0.058	0.080	0.063	0.070	0.054	0.072	0.042	0.080	0.128	0.107	0.046	0.205	0.052	0.111	0.095
GreyHornedHeathen	0.122	0.156	0.112	0.109	0.116	0.162	0.109	0.138	0.122	0.120	0.155	0.125	0.135	0	0.124	0.118	0.105	0.135	0.117	0.127	0.106	0.130	0.096	0.133	0.184	0.146	0.105	0.313	0.114	0.159	0.080
HungarianMerino	0.071	0.098	0.047	0.045	0.050	0.100	0.053	0.040	0.063	0.065	0.098	0.094	0.068	0.124	0	0.064	0.058	0.080	0.043	0.055	0.050	0.036	0.038	0.076	0.117	0.099	0.042	0.184	0.035	0.103	0.087
Laticauda	0.052	0.093	0.049	0.047	0.052	0.091	0.042	0.069	0.053	0.044	0.088	0.083	0.062	0.118	0.064	0	0.039	0.063	0.053	0.061	0.041	0.066	0.029	0.062	0.106	0.092	0.037	0.190	0.043	0.091	0.074
Leccese	0.045	0.088	0.044	0.042	0.048	0.081	0.038	0.064	0.049	0.037	0.079	0.078	0.058	0.105	0.058	0.039	0	0.056	0.048	0.057	0.036	0.061	0.024	0.056	0.099	0.085	0.032	0.185	0.038	0.084	0.063
Massese	0.067	0.111	0.067	0.066	0.071	0.110	0.062	0.089	0.072	0.068	0.105	0.098	0.080	0.135	0.080	0.063	0.056	0	0.072	0.080	0.060	0.085	0.049	0.081	0.128	0.104	0.055	0.213	0.064	0.110	0.093
Merinizzata	0.057	0.086	0.045	0.043	0.048	0.088	0.046	0.050	0.057	0.057	0.088	0.082	0.063	0.117	0.043	0.053	0.048	0.072	0	0.054	0.040	0.043	0.031	0.069	0.107	0.086	0.033	0.193	0.034	0.088	0.074
Merinolandschaf	0.067	0.099	0.052	0.049	0.054	0.099	0.051	0.063	0.062	0.065	0.098	0.093	0.070	0.127	0.055	0.061	0.057	0.080	0.054	0	0.050	0.057	0.036	0.078	0.119	0.098	0.045	0.199	0.043	0.104	0.086
Ojalada	0.048	0.082	0.036	0.035	0.040	0.081	0.028	0.056	0.037	0.043	0.077	0.076	0.054	0.106	0.050	0.041	0.036	0.060	0.040	0.050	0	0.052	0.016	0.056	0.097	0.081	0.029	0.180	0.030	0.083	0.065
Rambouillet	0.073	0.102	0.049	0.045	0.051	0.103	0.055	0.044	0.065	0.069	0.103	0.097	0.072	0.130	0.036	0.066	0.061	0.085	0.043	0.057	0.052	0	0.039	0.081	0.123	0.102	0.043	0.199	0.037	0.106	0.089
Rasaaragonesa	0.038	0.072	0.024	0.022	0.028	0.069	0.018	0.043	0.029	0.032	0.066	0.066	0.042	0.096	0.038	0.029	0.024	0.049	0.031	0.036	0.016	0.039	0	0.044	0.086	0.073	0.018	0.168	0.017	0.071	0.052
SardinianWhite	0.070	0.109	0.065	0.062	0.068	0.109	0.059	0.085	0.067	0.061	0.106	0.101	0.080	0.133	0.076	0.062	0.056	0.081	0.069	0.078	0.056	0.081	0.044	0	0.127	0.107	0.054	0.209	0.058	0.112	0.095
Schoonebeker	0.114	0.147	0.103	0.100	0.107	0.103	0.100	0.128	0.112	0.111	0.106	0.122	0.128	0.184	0.117	0.106	0.099	0.128	0.107	0.119	0.097	0.123	0.086	0.127	0	0.135	0.097	0.302	0.104	0.091	0.126
ScottishBlackface	0.095	0.121	0.091	0.088	0.095	0.119	0.084	0.108	0.095	0.097	0.115	0.100	0.107	0.146	0.099	0.092	0.085	0.104	0.086	0.098	0.081	0.102	0.073	0.107	0.135	0	0.081	0.234	0.088	0.118	0.104
Sopravissana	0.044	0.081	0.033	0.030	0.036	0.079	0.031	0.047	0.042	0.042	0.076	0.075	0.046	0.105	0.042	0.037	0.032	0.055	0.033	0.045	0.029	0.043	0.018	0.054	0.097	0.081	0	0.178	0.024	0.081	0.062
SpanishMerinosCordoba	0.202	0.229	0.183	0.180	0.187	0.276	0.183	0.205	0.192	0.194	0.272	0.226	0.205	0.313	0.184	0.190	0.185	0.213	0.193	0.199	0.180	0.199	0.168	0.209	0.302	0.234	0.178	0	0.188	0.279	0.279
SpanishMer.EstremaDura	0.052	0.084	0.031	0.028	0.034	0.085	0.032	0.043	0.044	0.046	0.083	0.080	0.052	0.114	0.035	0.043	0.038	0.064	0.034	0.043	0.030	0.037	0.017	0.058	0.104	0.088	0.024	0.188	0	0.088	0.067
VeluweHeathen	0.097	0.128	0.089	0.086	0.094	0.099	0.086	0.113	0.097	0.097	0.093	0.104	0.111	0.159	0.103	0.091	0.084	0.110	0.088	0.104	0.083	0.106	0.071	0.112	0.091	0.118	0.081	0.279	0.088	0	0.104
WhiteHornedHeathen	0.081	0.115	0.072	0.067	0.075	0.107	0.067	0.097	0.078	0.078	0.102	0.082	0.095	0.080	0.087	0.074	0.063	0.093	0.074	0.086	0.065	0.089	0.052	0.095	0.126	0.104	0.062	0.279	0.067	0.104	0
