# Supplementary material for: Position of Hungarian Merino among other Merinos, within-breed genetic similarity network and markers associated with daily weight gain
Source: Anim Biosci. 2022 Jun 24;36(1):10–8. doi: 10.5713/ab.21.0459 (PMC9834658; doi:10.5713/ab.21.0459)
Supplement: Supplementary file 3 [file ab-21-0459-suppl3.pdf]

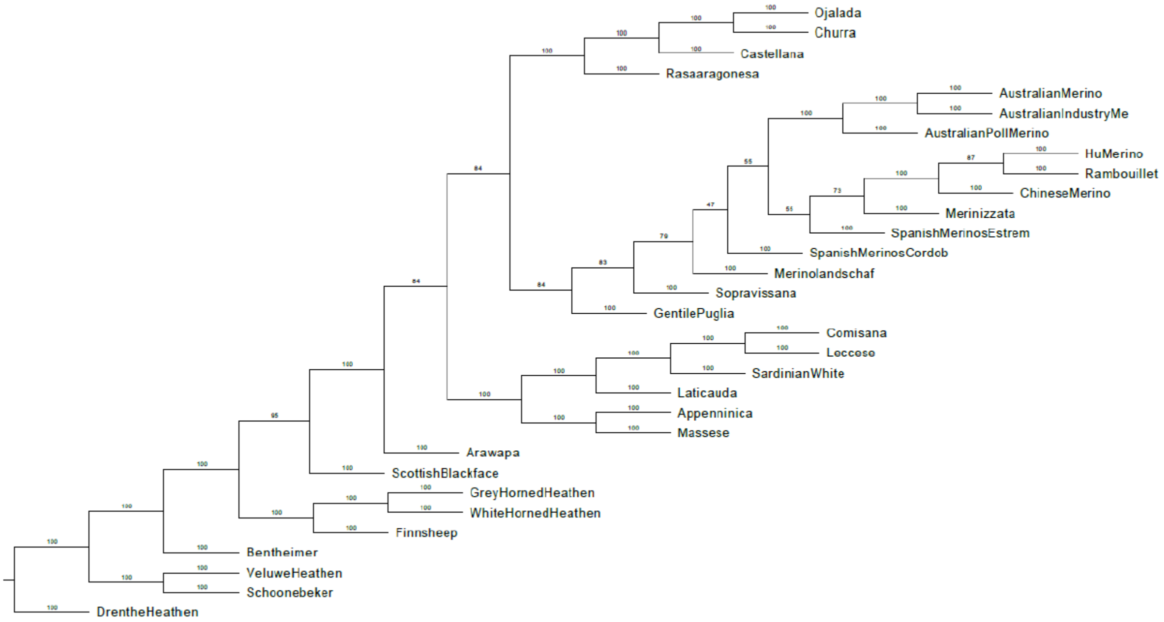

Supplementary Figure S2.

UPGMA tree of Nei's genetic distance sheep breeds. Bootstrap values are indicated on the nodes.
