# Supplementary material for: Position of Hungarian Merino among other Merinos, within-breed genetic similarity network and markers associated with daily weight gain
Source: Anim Biosci. 2022 Jun 24;36(1):10–8. doi: 10.5713/ab.21.0459 (PMC9834658; doi:10.5713/ab.21.0459)
Supplement: Supplementary file 2 [file ab-21-0459-suppl2.pdf]

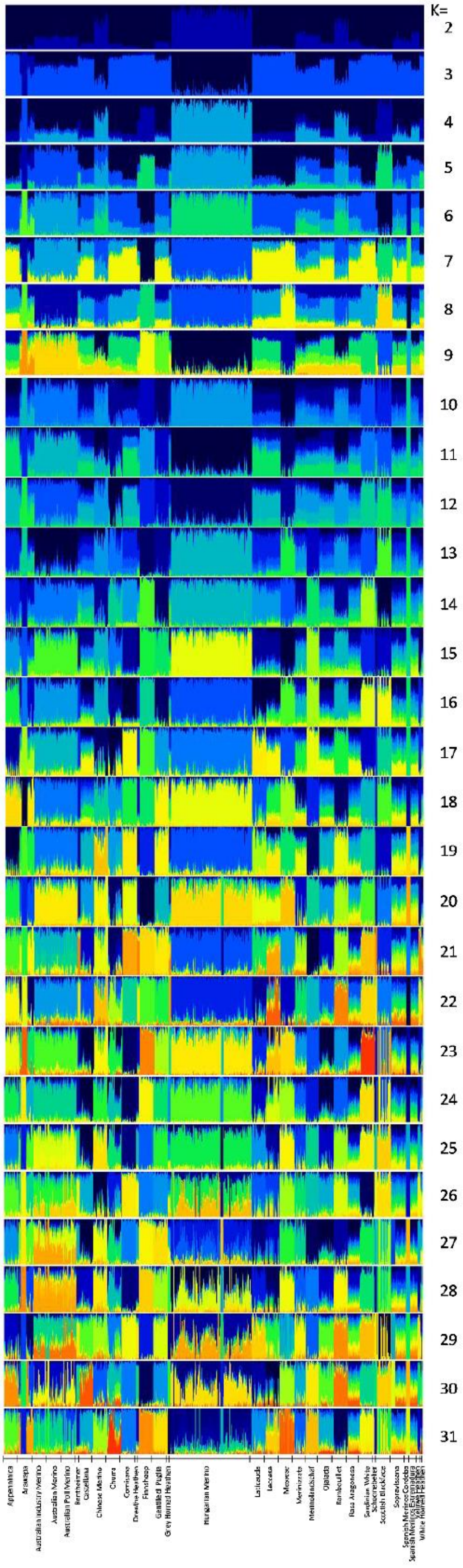

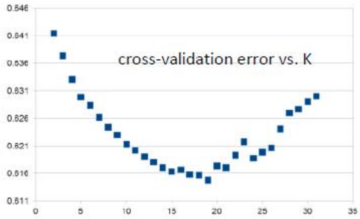

Supplementary Figure S1.

Admixture analysis of 31 Merino-derived breeds and for a range of K-values (K=2 to 31) and cross-validation error at different K values. Each individual is represented by a single column divided into K coloured segments, where K is the number of assumed clusters.
